# Association between obesity and subsequent risk of myasthenia gravis: a nationwide population-based cohort study

**DOI:** 10.1007/s13760-026-03022-y

**Published:** 2026-03-13

**Authors:** Soonwook Kwon, Ye Bin Park, Kyung-Do Han, Yeon Hak Chung, Eun Bin Cho, Dong Wook Shin, Ju-Hong Min

**Affiliations:** 1 Department of Neurology, Inha University Hospital, Inha University College of Medicine, Incheon, South Korea; 2 Department of Statistics and Actuarial Science, Soongsil University, Seoul, South Korea; 3 Department of Neurology, Korea University Guro Hospital, University College of Medicine, Seoul, South Korea; 4 Department of Neurology, College of Medicine, Gyeongsang Institute of Health Science, Gyeongsang National University, Jinju, South Korea; 5 Department of Neurology, Gyeongsang National University Changwon Hospital, Changwon, South Korea; 6 Department of Family Medicine & Supportive Care Center, Samsung Medical Center, Sungkyunkwan University School of Medicine, Seoul, South Korea; 7 Department of Clinical Research Design and Evaluation, Samsung Advanced Institute of Health Sciences & Technology, Sungkyunkwan University, Seoul, South Korea; 8 Department of Digital Health, Samsung Advanced Institute of Health Sciences & Technology, Sungkyunkwan University, Seoul, South Korea; 9 Department of Neurology, Samsung Medical Center, Sungkyunkwan University School of Medicine, Seoul, South Korea; 10 Neuroscience Center, Samsung Medical Center, Seoul, South Korea; 11 Department of Health Sciences and Technology, Samsung Advanced Institute for Health Sciences & Technology, Sungkyunkwan University, Seoul, South Korea

**Keywords:** Myasthenia gravis, Obesity, Epidemiology, Risk factor, Cohort study

## Abstract

**Background:**

Obesity causes a pro-inflammatory environment and is an established risk factor for a variety of autoimmune diseases. However, the association with the subsequent risk of myasthenia gravis (MG) was unclear.

**Methods:**

This nationwide cohort study evaluated 3,937,214 adults over 20 years old, using data from the Korean National Health Insurance Service. Participants were categorized by body mass index (BMI) into underweight (BMI < 18.5), normal (18.5 ≤ BMI < 23.0), preobese (23.0 ≤ BMI < 25.0), obese class I (25.0 ≤ BMI < 30.0), and obese class II groups (≥ 30.0). The primary outcome was newly diagnosed MG during the follow-up period, identified through the International Classification of Diseases-10th revision, diagnostic codes and enrollment in the Rare Intractable Diseases program of South Korea. Cox proportional hazards models were used to calculate adjusted hazard ratios (aHRs) for MG risk by BMI.

**Results:**

During about 10-year follow-up, 799 individuals developed MG (mean age 47.3 years old [SD, 14.02 years], male 54.6%, and BMI ≥ 25 kg/m^2^ 32.6%). The incidence rate was highest in the obese class II group (3.39/100,000 person-years). Obese class I (aHR 1.20, 95% CI 1.00–1.43) and class II (aHR 1.88, 95% CI 1.37–2.56) groups demonstrated significantly elevated MG risks compared to the normal BMI group. In subgroup analyses, underweight males had a notably increased risk (aHR 2.16, 95% CI 1.24–3.78).

**Conclusion:**

Obesity has an association with an increased risk of subsequent MG, suggesting a potential risk factor of MG. Further studies are needed to elucidate the association between obesity and MG.

**Supplementary Information:**

The online version contains supplementary material available at 10.1007/s13760-026-03022-y.

## Introduction

Myasthenia gravis (MG) is a rare autoimmune disease of neuromuscular junctions characterized by muscle weakness and fluctuations in repetitive muscle activity [1]. Anti-acetylcholine receptor antibodies are identified in approximately 80% of cases of MG and are pathognomonic. Antibody production due to loss of self-tolerance is caused by loss of tolerance to the acetylcholine receptor during lymphocyte maturation in the presence of thymus hyperplasia or thymoma. Genetic risk factors, such as human leukocyte antigen (HLA) genes and non-HLA genes including *PTPN22* and *CTLA4*, are known to contribute to the development of MG, but non-genetic risk factors except thymic disorders remain unknown [2]. However, recently several population-based studies have suggested associations between MG risk and lifestyle or environmental factors, including smoking, socioeconomic status, alcohol consumption, and hormonal states such as the postpartum period [3–6]. In addition, a Chinese case-control study found that diabetes increased the risk of MG by about 1.9-fold, especially among women and older adults [7].

Obesity is a modifiable risk factor that induces a pro-inflammatory state and dysregulates the balance of helper T_17_ and regulatory T cells, thereby increasing the risk of developing various autoimmune diseases such as rheumatoid arthritis, multiple sclerosis, type 1 diabetes, psoriasis, and Hashimoto thyroiditis [8]. In a previous study of 40 adult patients with MG in Sweden, patients with late-onset MG were more obese than their matched controls (odds ratio 13.7, 95% confidence interval [CI] 1.7–112.9). In addition, a nationwide Swedish cohort study demonstrated that obesity was independently associated with severe generalized MG, as reflected by higher MG-ADL scores [9]. However, the number of patients with MG was small in the case-control study and the cross-sectional nature of existing data, the role of obesity as a predisposing risk factor for MG in the general population remains unclear.

In the present study, we evaluated the association between obesity and the subsequent risk of MG in adults using a nationwide population cohort from the Korean National Health Insurance Service (KNHIS) database.

## Methods and materials

### Data source

This retrospective nationwide population-based cohort study was conducted using the KNHIS database, which draws from the mandatory health insurance system operated by the Ministry of Health and Welfare of South Korea and covers more than 97% of the population [10]. The KNHIS database includes demographic information such as age, sex, income level, and region and also provides claims data with diagnosis codes defined according to the International Classification of Diseases, 10th Revision (ICD-10). In addition, a biennial national health screening program is offered to all beneficiaries over the age of 40 and all company workers over the age of 20, with eligibility maintained throughout the lifespan [11]. Health screening data are curated in the KNHIS database, including anthropometric measurements (body weight, height, and waist circumference), laboratory tests (blood glucose, lipid profile, serum creatinine, liver function test, and urine analysis) and health questionnaires to determine lifestyle factors (comorbidities, smoking status, alcohol consumption, physical activity, and family history) [12]. Furthermore, the KNHIS links to a death registry database to manage the qualification of enrollees.

This research was reviewed and approved by the Institutional Review Board of Samsung Medical Center (IRB No. 2025-04-084). The requirement for informed consent was waived by the board because we used de-identified retrospective data.

### Study population

**Fig. 1:**
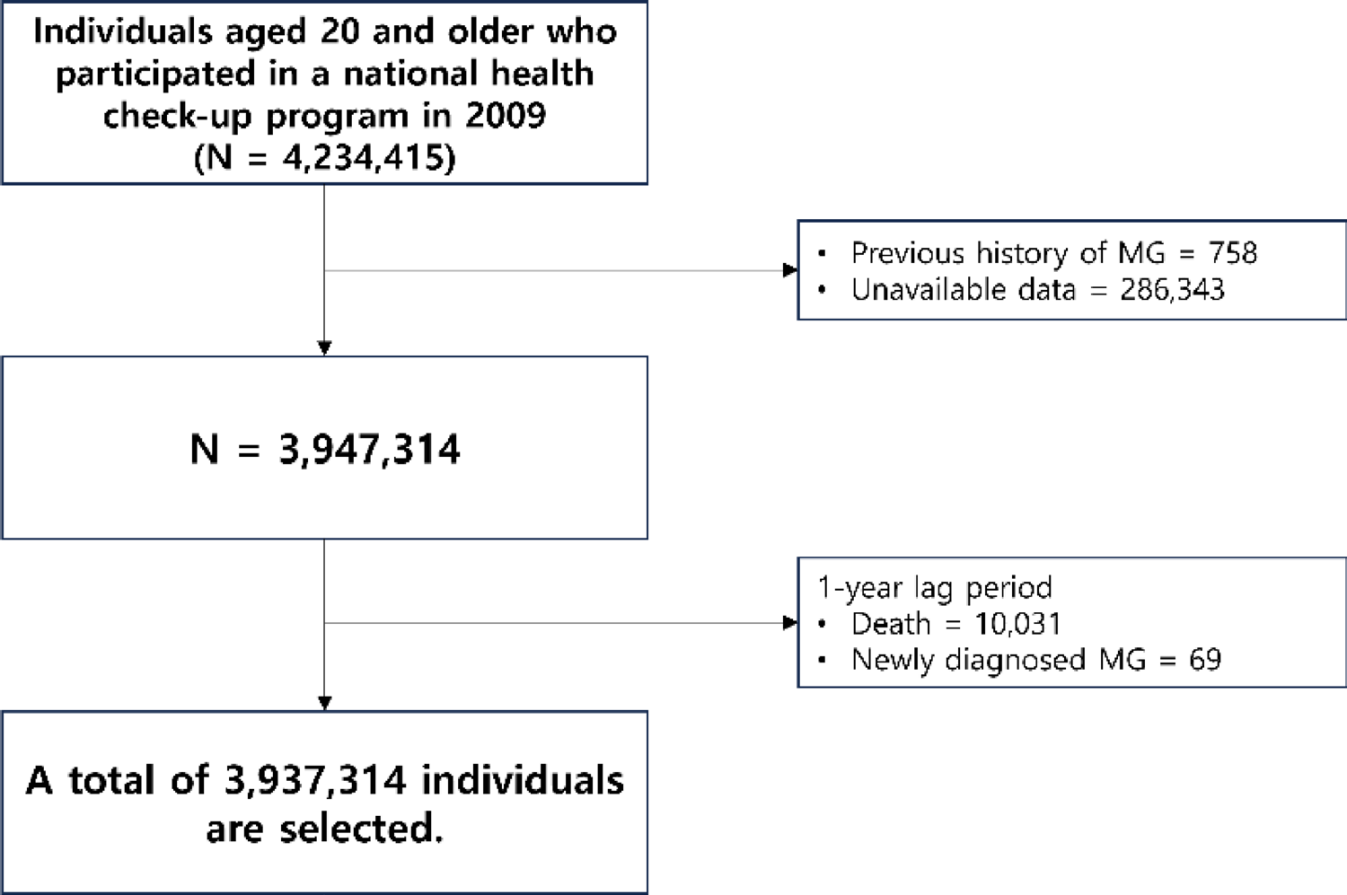
Flow chart of study population enrollment

We reviewed individuals in the KNHIS database who were 20 years of age or older and were part of the national health screening program in 2009. We were able to access data for a total of 4,234,415 individuals due to the personal information protection policy of KNHIS. Among those, we excluded individuals who had a history of MG (*N* = 758) and incomplete medical information (*N* = 286,343). In addition, individuals who were diagnosed with MG (*N* = 69) or had a documented death for any reason (*N* = 10,031) within one year after national health screening program were also excluded. After the inclusion and exclusion criteria were followed, 3,937,214 individuals were identified. We followed this cohort from January 2009 to December 2020. Censoring was applied if MG was diagnosed, or death occurred during the follow-up period. The flowchart of study population enrollment is described in Fig. 1.

### Definition of myasthenia gravis

The primary outcome of this study was the newly diagnosed MG during the follow-up period defined as meeting the following criteria: (1) ≥ 2 outpatient or ≥ 1 hospitalization claims with the ICD-10 code (G70.0) and (2) registration in the Rare Intractable Diseases (RID) program with the code for MG (V012). The RID program has provided additional medical payment support to individuals with rare and intractable diseases since 2009, and registered individuals pay only 10% of their medical expenses out of pocket. Enrollment in the RID program is made by neurologists based on clinical symptoms, serologic testing for antibodies (against acetylcholine receptor and muscle-specific kinase), and the results of repetitive nerve stimulation tests.

### Grouping by body mass index

Body mass index (BMI, kg/m^2^) was calculated using body weight and height measured at the time of the health screening program in 2009. Based on the World Health Organization’s recommendations for the Asia-Pacific region, we divided the population into five subgroups according to BMI (kg/m^2^) [13]: underweight (BMI < 18.5), normal (18.5 ≤ BMI < 23.0), preobese (23.0 ≤ BMI < 25.0), obese class I (25.0 ≤ BMI < 30.0), and obese class II groups (≥ 30.0) (Table 1).

**Table 1:** Body mass index (kg/m^2^) classification following the World Health Organization’s recommendations

Classification	BMI in the Asia-Pacific region	BMI outside the Asia-Pacific region
Underweight	< 18.5	< 18.5
Normal range	18.5–22.9	18.5–24.9
Overweight	≥ 23.0	≥ 25.0
Preobese	23.0–24.9	25.0–29.9
Obese class I	25.0–29.9	30.0–34.9
Obese class II	≥ 30.0	35.0–39.9
Obese class III		≥ 40.0

In addition, abdominal obesity was assessed using waist circumference (≥ 90 cm in male and ≥ 85 cm in female) [14], and additional analyses were performed based on this definition.

### Demographic, social, and comorbidity variables

Smoking status was classified as current (≥ 100 cigarettes in lifetime and continuing), ex-smoker (≥ 100 cigarettes in lifetime, but quit), or non-smoker (< 100 cigarettes in lifetime). Alcohol consumption was classified as heavy drinkers (≥ 30 g/day for male and ≥ 20 g/day for female), mild drinkers (< 30 g/day for male and < 20 g/day for female), and non-drinkers. Regular exercise was defined as at least 20 min of vigorous physical activity ≥ 3 days per week or ≥ 30 min of moderate-intensity physical activity ≥ 5 days per week. Low income was defined as the bottom 25% of insurance premium paid and medical aid beneficiaries, as a proxy for income status.

Comorbidity information was obtained from health questionnaires for lifestyle factors or health screening program data [15]. Diabetes mellitus was defined as one or more claims per year under ICD-10 codes E10–14 and one or more claims per year for prescription of anti-diabetic medication. Hypertension was defined as one or more claims per year for ICD-10 codes I10–13, or I15 and one or more claims per year for a prescription of antihypertensive medication. Dyslipidemia was defined as one or more claims per year under ICD-10 code E78 and one or more claims per year for prescription of a lipid-lowering medication. Thymic disorders defined as a diagnosis of one of the following: malignancy, benign neoplasm, or thymic hyperplasia (ICD-10 codes C37, D15.0, and E32.0, respectively) from the KNHIS database.

### Statistical analysis

Data are described as mean ± standard deviation (SD) for continuous variables and number (percentage) for categorical variables. When comparing two groups, we used Student’s t-test or analysis of variance for continuous variables and the chi-square for categorical variables. The incidence rate of MG was estimated as the number of events per 100,000 person-years. The Kaplan-Meier method and the log-rank test were used to compare the cumulative incidence rates of MG between groups. The crude hazard ratios (HRs) and adjusted hazard ratios (aHRs) with 95% CI were estimated using Cox proportional hazards models based on BMI. In the adjusted model, age, sex, income status, smoking status, alcohol consumption, regular exercise, and presence of diabetes mellitus, hypertension, dyslipidemia, and thymic disorders were used as potential confounders.

Subgroup analyses were performed according to age (< 65 and ≥ 65) and sex. The statistical significance of the interaction was assessed using a Wald test for the interaction term included in the model. All statistical analyses were conducted using SAS statistical package version 9.4 (SAS Institute Inc., Cary, NC, USA), and *P* < 0.05 was considered statistically significant.

## Results

During the follow-up period of 10.1 ± 1.27 years, a total of 799 cases of MG were newly diagnosed among 3,937,214 individuals (Table 2). The mean age of the study population was 47.3 ± 14.02 years, 2,259,974 (57.4%) were aged 20–49 years, and 2,148,184 (54.6%) were male. Mean BMI was 23.7 ± 3.23, and 1,285,228 individuals (32.6%) had BMI ≥ 25 kg/m^2^. Thymic disorders were present in 495 patients (0.01%) with no difference between groups. The higher BMI group has higher rates of current smokers and heavy alcohol consumption, and higher prevalence of diabetes mellitus, hypertension, and dyslipidemia. In laboratory data, the mean systolic and diastolic blood pressure, fasting glucose, total cholesterol, low-density lipoprotein cholesterol, and geometric means of triglyceride were increased in the high BMI group and high-density lipoprotein cholesterol decreased with increasing BMI. Baseline characteristics including demographic, comorbidities, and laboratory data are described in Table 2.

**Table 2:** Baseline characteristics of the study population

	Total population	Underweight	Normal	Preobese	Obese class I	Obese class II	*P*-value
	*N* = 3,937,214 (100%)	*N* = 145,599 (3.70%)	*N* = 1,536,457 (39.0%)	*N* = 969,930 (24.6%)	*N* = 1,145,391 (29.1%)	*N* = 139,837 (3.6%)	
***Demographics***							
Age, years	47.3 ± 14.02	40.5 ± 16.61	45.4 ± 14.33	48.9 ± 13.31	49.3 ± 13.21	46.4 ± 13.86	< 0.001
20–49	2,259,974 (57.4)	107,822 (74.1)	970,744 (63.2)	510,321 (52.6)	587,608 (51.3)	83,479 (59.7)	< 0.001
50–64	1,160,906 (29.5)	18,967 (13.0)	385,848 (25.1)	325,125 (33.5)	391,797 (34.2)	39,169 (28.0)	
≥ 65	516,334 (13.1)	18,810 (12.9)	179,865 (11.7)	134,484 (13.9)	165,986 (14.5)	17,189 (12.3)	
Sex							
Male	2,148,184 (54.6)	48,211 (33.1)	724,017 (47.1)	578,289 (59.6)	719,654 (62.8)	78,013 (55.8)	< 0.001
Female	1,789,030 (45.4)	97,388 (66.9)	812,440 (52.9)	391,641 (40.4)	425,737 (37.2)	61,824 (44.2)	
Height, cm	163.8 ± 9.23	162.8 ± 8.12	163.3 ± 8.80	164.1 ± 9.31	164.4 ± 9.62	164.0 ± 10.60	< 0.001
Weight, kg	63.9 ± 11.62	46.7 ± 5.16	56.6 ± 7.00	64.7 ± 7.48	72.5 ± 9.13	86.4 ± 12.31	< 0.001
BMI, kg/m^2^	23.7 ± 3.23	17.6 ± 0.78	21.1 ± 1.21	23.9 ± 0.57	26.7 ± 1.29	32.0 ± 2.25	< 0.001
Waist circumference, cm	80.2 ± 9.11	66.2 ± 5.71	74.3 ± 6.31	81.2 ± 5.63	87.2 ± 6.17	96.8 ± 7.79	< 0.001
Smoking							
None	2,347,903 (59.6)	102,507 (70.4)	984,643 (64.1)	554,872 (57.2)	625,673 (54.6)	80,208 (57.4)	< 0.001
Ex	564,707 (14.3)	10,035 (6.9)	171,514 (11.2)	160,266 (16.5)	204,093 (17.8)	18,799 (13.4)	
Current	1,024,604 (26.0)	33,057 (22.7)	380,300 (24.8)	254,792 (26.3)	315,625 (27.6)	40,830 (29.2)	
Alcohol consumption							
None	2,035,387 (51.7)	82,724 (56.8)	823,175 (53.6)	490,630 (50.6)	565,883 (49.4)	72,975 (52.2)	< 0.001
Mild	1,565,992 (39.8)	54,688 (37.6)	604,724 (39.4)	394,580 (40.7)	460,690 (40.2)	51,310 (36.7)	
Heavy	335,835 (8.5)	8,187 (5.6)	108,558 (7.1)	84,720 (8.7)	118,818 (10.4)	15,552 (11.1)	
Regular exercise	707,730 (18.0)	14,066 (9.7)	250,959 (16.3)	192,920 (19.9)	225,870 (19.7)	23,915 (17.1)	< 0.001
Low income level	766,572 (19.5)	32,660 (22.4)	319,178 (20.8)	180,148 (18.6)	206,738 (18.1)	27,848 (19.9)	< 0.001
***Comorbidities***							
Diabetes mellitus	267,731 (6.8)	3,655 (2.5)	68,680 (4.5)	68,477 (7.1)	107,781 (9.4)	17,689 (12.7)	< 0.001
Hypertension	816,578 (20.7)	10,702 (7.4)	199,432 (13)	211,736 (21.8)	339,494 (29.6)	50,761 (36.3)	< 0.001
Dyslipidemia	522,075 (13.3)	5,504 (3.8)	131,982 (8.6)	138,700 (14.3)	212,699 (18.6)	30,722 (22.0)	< 0.001
Thymic disorders	495 (0.01)	13 (0.01)	157 (0.01)	135 (0.01)	168 (0.01)	22 (0.02)	0.28

BMI, body mass index

### Risks of myasthenia gravis according to body mass index

**Fig. 2:**
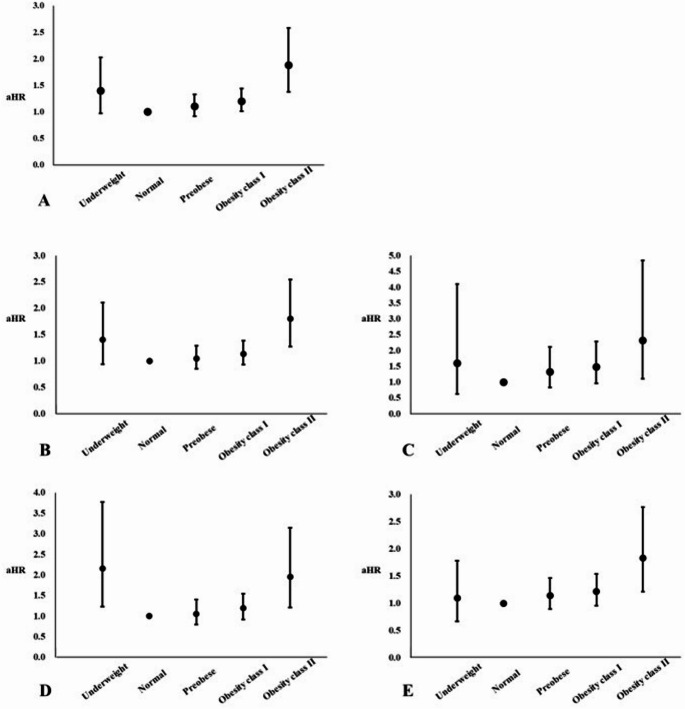
The risk of myasthenia gravis (adjusted hazard ratios)^*^ based on body mass index. (**A**) Total study population. (**B**) 20–64 years old. (**C**) 65 years of age or older. (**D**) Male. (**E**) Female. ^*^ The adjusted hazard ratios with 95% CI were estimated using Cox proportional hazards models based on BMI. Potential confounders included age, sex, income status, smoking status, alcohol consumption, regular exercise, and presence of diabetes mellitus, hypertension, dyslipidemia, and thymic disorders

**Fig. 3:**
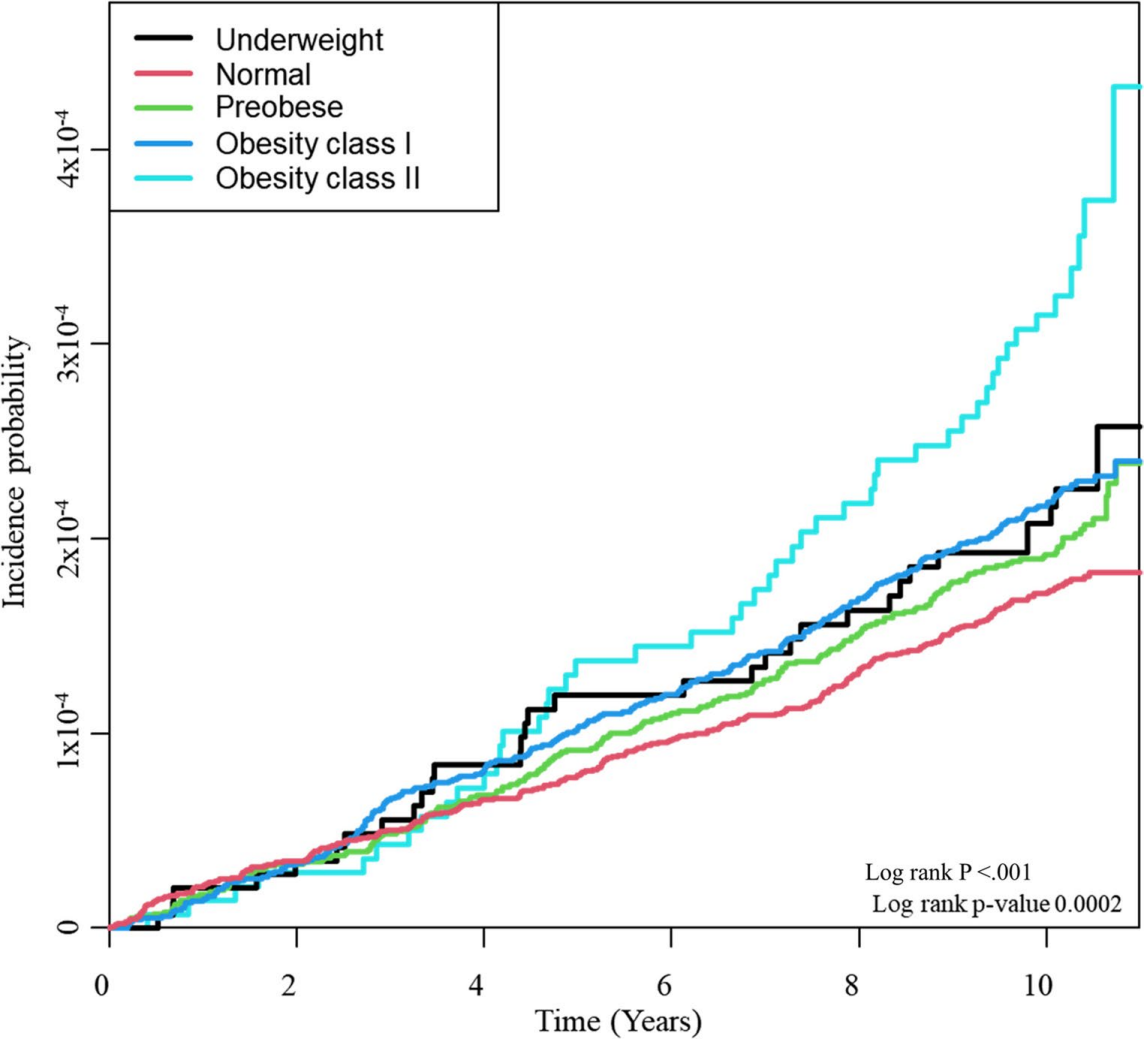
Kaplan–Meier curves of the incidence probability of myasthenia gravis

The incidence rate of MG was 2.01 per 100,000 person-years in the present study: 3.39/100,000 person-years in the obese class II group, 2.20 in the obese class I group, 1.99 in the preobese group, 1.73 in the normal BMI group, and 2.22 in the underweight group. Obese class I and II groups had significantly higher risks of MG compared with the normal BMI group (aHR 1.20, 95% CI 1.00–1.43 and aHR 1.88, 95% CI 1.37–2.56, respectively) after adjustment for age, sex, low income, smoking, alcohol consumption, regular exercise, diabetes mellitus, hypertension, dyslipidemia, and thymic disorder (Table 3; Fig. 2A). The aHRs of the preobese and underweight groups were higher compared with the normal BMI group, but those were not significant (aHR 1.10, 95% CI 0.91–1.32 and aHR 1.40, 95% CI 0.97–2.03, respectively). The Kaplan-Meier curve showed an increase in the cumulative incidence of MG over the follow-up period in all other groups compared with normal BMI group (Fig. 3).

**Table 3:** Crude and adjusted hazard ratios of myasthenia gravis according to body mass index (BMI)

BMI (kg/m^2^)	*N*	Myasthenia gravis	Duration, years	Incidence rate, per 100,000 person-years	Hazard ratio (95% CI)	
					Crude	Adjusted^*^
Underweight	145,599	32	1,440,757.7	2.22	1.29 (0.89–1.86)	1.40 (0.97–2.03)
Normal	1,536,457	268	15,520,320.8	1.73	1 (Ref.)	1 (Ref.)
Preobese	969,930	196	9,827,187.9	1.99	1.15 (0.96–1.39)	1.10 (0.91–1.32)
Obese class I	1,145,391	255	11,611,252.0	2.20	1.27 (1.07–1.51)	1.20 (1.00–1.43)
Obese class II	139,837	48	1,416,886.4	3.39	1.96 (1.44–2.67)	1.88 (1.37–2.56)
Total	3,937,214	799	39,816,404.8	2.01		

* Adjusted for age, sex, low income, smoking, alcohol consumption, regular exercise, presence of diabetes mellitus, hypertension, dyslipidemia, and thymic disorders

Abdominal obesity (waist circumference ≥ 90 cm in male and ≥ 85 cm in female) was associated with an increased risk of MG (aHR 1.33, 95% CI 1.15–1.54; Supplementary Table 2). The incidence of MG increased progressively with higher waist circumference categories.

### Subgroup analyses stratified by age and sex

There were no significant modifying effects of age or sex on the risk of MG by BMI in any subgroups (Fig. 2B-E and Supplementary Table 1). In all subgroups, the obese class II had an association with the increased subsequent risk of MG and, only in the male subgroup was the underweight group associated with an increased risk of MG.

## Discussion

In the present study, we demonstrated that obese individuals had an increased risk of subsequent MG compared with those without obesity. Compared with the normal BMI group, the risk of subsequent MG increased with increasing BMI, and was 1.88 times higher for individuals in the obese class II group. A similar pattern was observed when abdominal obesity was assessed using waist circumference, with the risk of MG increasing across higher waist circumference categories.

Obese individuals had approximately 1.20 to 1.88 times the risk of MG compared to those with normal BMI. Previously, an epidemiologic study from Denmark followed up a cohort of 75,008 young women of reproductive age (median age 30.2 years, interquartile range 27.4–33.3) for a mean of 11.4 years, with a total of 13 cases of MG defined by the ICD codes (ICD-8 code 733.09 and ICD-10 code G70.0). In that study, they found that the HR for MG was 0.84 (95% CI 0.69–1.02) per unit increase in BMI, which was not consistent with our results [16]. However, only young women were included in that cohort, and given that MG is a rare disease with an annual incidence of 1.34 per 100,000 in Denmark [17], the size of cohort is small to analyze statistically. In our study, the incidence of MG (2.01/100,000 person-years) was similar to previous reports in South Korea (1.18–2.44/100,000 person-years) [18, 19], and we analyzed a cohort of 4 million individuals to achieve sufficient statistical significance. In addition, BMI was defined by the World Health Organization’s recommendations for the Asia-Pacific region, which is appropriate to determine the impact of obesity on MG in the Asian population.

A similar effect size for the association between obesity and disease risk has been reported in other autoimmune diseases. Previous epidemiologic studies and recent meta-analyses have shown that obesity is associated with an approximately 20–30% increased risk of rheumatoid arthritis, a 40–50% increased risk of multiple sclerosis, and a 30–80% increased risk of psoriasis compared with the normal BMI group [20–23].

There are several possible explanations that obesity increases the risk of MG by creating a proinflammatory environment. First, adipokines secreted by adipose cells play an important role in the development of autoimmune diseases [8]. Leptin, which is increased in obesity, inhibits the differentiation and proliferation of regulatory T cells [24] and promotes memory T cells to differentiated into helper T_1_ or T_17_ cells [25]. In addition, the plasma level of adiponectin, which has anti-inflammatory activity, decreased in obese individuals and similar findings were observed in MG patients [26, 27]. Second, apoptosis inhibitor of macrophage, increased in serum under obese conditions, induced the infiltration of proinflammatory M1-macrophage in adipose tissue and production of immunoglobulin G autoantibodies [8, 28]. Third, vitamin D deficiency, commonly accompanied with obesity [29], also increases helper T_1_ and T_17_ cells differentiation and inhibits regulatory T cells [30]. A recent systematic review revealed that there were significantly lower levels of vitamin D in MG patients than in normal controls [31]. However, it is uncertain whether obesity directly affects the development of MG or whether common causative factors increase the risk of obesity and MG, simultaneously, which is needed to be further elucidated.

In the other groups, the slopes of incidence of probability in Kaplan-Meier’s curve were relatively constant over the follow-up period, but that in the Obesity Class II group rose much more steeply with longer follow-up. This suggests that the effect of obesity on the occurrence of MG may be cumulative. In addition, it may be related to the fact that BMI of obese individuals tend to increase with aging [32], whereas it has been reported that BMI typically increases until age 60 and then decreases thereafter [33].

In the subgroup analyses, age and sex did not have a modifying effect on the risk of MG by BMI, with similar trends to the results in the overall population. In all subgroups, the aHR for the risk of MG increased with higher BMI, and the obese class II group had a significantly higher risk of MG than the normal BMI group. Male individuals with underweight had an increased risk of MG, which still remained significant after adjusting for covariates. The association of underweight with autoimmune diseases is not well understood, but increased risk and higher mortality in neuromyelitis optic spectrum disorder, rheumatoid arthritis, and Crohn’s disease have been reported in underweight individuals [16, 34, 35]. Underweight can also cause dysregulation of the immune system [36], which may increase the risk of MG, but can be caused by bulbar symptoms of MG before the diagnosis of MG [1, 37].

### Strength and clinical implications

The present study had several notable strengths and clinical implications. To our knowledge, our study is the first large cohort study to demonstrate the association between BMI and the subsequent risk of MG in the general population. The inclusion of a large population-based cohort with an age distribution representative of the general adult population at the time of data collection [38] and adjustment for multiple variables in the analysis of MG risk strengthens the reliability of our findings. Furthermore, the obesity criteria was based on the WHO Asian-Pacific region criteria [13], which makes it more generalizable to the Asian population. Our results suggest that obesity might be one of the potential risk factors for MG in the Korean populations, although the causative relationship between obesity and MG remains to be further elucidated.

### Limitations

This study also had severe limitations. First, obesity was assessed by BMI, and data on body composition and fat distribution were not analyzed. The distribution of muscle and fat influences the development of autoimmune diseases because adipose cells in fat provoke a pro-inflammatory environment [8], and skeletal muscle has anti-inflammatory effects by regulating immune system function [39]. However, we analyzed the association between obesity and MG by adjusting for regular exercise which can affect body composition. Second, BMI was only measured and categorized at the start of follow-up, and trajectories of BMI were not considered. Third, since our data were claim-based, and did not give information on clinical presentation, including type or severity, the presence or titer of antibodies to acetylcholine receptor or muscle-specific kinase or genetic risk factors. Finally, although relatively small individuals (*N* = 286,343, 6.8%) were excluded due to incomplete medical information, this missing data might have influenced our results. However, the incidences of MG in South Korea have been reported to be low, ranging from 1.18 to 1.84 per 100,000 people [18], so missing data is unlikely to have a significant impact on the results.

## Conclusion

The present study showed that obesity is associated with the subsequent risk of MG and the risk of MG was more increased in those with higher BMI. Because the causal relationship between obesity and MG remains uncertain, further prospective and pathophysiologic studies are needed.

## Supplementary Information

Below is the link to the electronic supplementary material.

Supplementary Material 1

## Data Availability

Data are available upon reasonable request. The data set analyzed in this study is not publicly available because of restricted access, but further information about the data set is available from the corresponding author on reasonable request.

## References

[1] Gilhus NE, Tzartos S, Evoli A, Palace J, Burns TM, Verschuuren J (2019) Myasthenia gravis. Nat Rev Dis Primers 5:30 31048702 10.1038/s41572-019-0079-y

[2] Gilhus NE, Verschuuren JJ (2015) Myasthenia gravis: subgroup classification and therapeutic strategies. Lancet Neurol 14:1023–1036 26376969 10.1016/S1474-4422(15)00145-3

[3] Westerberg E, Landtblom AM, Punga AR (2018) Lifestyle factors and disease-specific differences in subgroups of Swedish Myasthenia Gravis. Acta Neurol Scand 138:557–565 30155967 10.1111/ane.13017

[4] Miyazaki Y, Sakushima K, Niino M, Takahashi E, Oiwa K, Naganuma R, Amino I, Akimoto S, Minami N, Yabe I, Kikuchi S (2023) Smoking and younger age at onset in anti-acetylcholine receptor antibody-positive myasthenia gravis. Immunol Med 46:77–83 36346077 10.1080/25785826.2022.2143077

[5] Boldingh MI, Maniaol AH, Brunborg C, Weedon-Fekjaer H, Verschuuren JJ, Tallaksen CM (2016) Increased risk for clinical onset of myasthenia gravis during the postpartum period. Neurology 87:2139–2145 27770065 10.1212/WNL.0000000000003339 PMC5109939

[6] Petersson M, Jons D, Feresiadou A, Ilinca A, Lundin F, Johansson R, Budzianowska A, Roos AK, Kagstrom V, Gunnarsson M, Sundstrom P, Klareskog L, Olsson T, Kockum I, Piehl F, Alfredsson L, Brauner S (2025) Nicotine, Alcohol Consumption, and Risk of Myasthenia Gravis: Results From the Swedish Nationwide GEMG Study. Neurology 105:e213771 40493875 10.1212/WNL.0000000000213771 PMC12165285

[7] Liu YD, Tang F, Li XL, Liu YF, Zhang P, Yang CL, Du T, Li H, Wang CC, Liu Y, Yang B, Duan RS (2023) Type 2 diabetes mellitus as a possible risk factor for myasthenia gravis: a case-control study. Front Neurol 14:1125842 37139075 10.3389/fneur.2023.1125842 PMC10149973

[8] Versini M, Jeandel PY, Rosenthal E, Shoenfeld Y (2014) Obesity in autoimmune diseases: not a passive bystander. Autoimmun Rev 13:981–1000 25092612 10.1016/j.autrev.2014.07.001

[9] Petersson M, Feresiadou A, Jons D, Ilinca A, Lundin F, Johansson R, Budzianowska A, Roos AK, Kagstrom V, Gunnarsson M, Sundstrom P, Piehl F, Brauner S (2021) Patient-Reported Symptom Severity in a Nationwide Myasthenia Gravis Cohort: Cross-sectional Analysis of the Swedish GEMG Study. Neurology 97:e1382–e1391 34376512 10.1212/WNL.0000000000012604 PMC8520390

[10] Shin DW, Cho B, Guallar E (2016) Korean National Health Insurance Database. JAMA Intern Med 176:138 26747667 10.1001/jamainternmed.2015.7110

[11] Shin DW, Cho J, Park JH, Cho B (2022) National General Health Screening Program in Korea: history, current status, and future direction. Precis Future Med 6:9–31

[12] Jeong SM, Lee HR, Han K, Jeon KH, Kim D, Yoo JE, Cho MH, Chun S, Lee SP, Nam KW, Shin DW (2022) Association of Change in Alcohol Consumption With Risk of Ischemic Stroke. Stroke 53:2488–2496 35440171 10.1161/STROKEAHA.121.037590

[13] Organization WH (2000) The Asia-Pacific perspective. redefining obesity and its treatment

[14] Lee SY, Park HS, Kim DJ, Han JH, Kim SM, Cho GJ, Kim DY, Kwon HS, Kim SR, Lee CB, Oh SJ, Park CY, Yoo HJ (2007) Appropriate waist circumference cutoff points for central obesity in Korean adults. Diabetes Res Clin Pract 75:72–80 16735075 10.1016/j.diabres.2006.04.013

[15] Kwon S, Kim B, Han KD, Jung W, Cho EB, Yang JH, Shin DW, Min JH (2023) Increased risk of myocardial infarction in amyotrophic lateral sclerosis: A nationwide cohort study in South Korea. J Neurol Sci 454:120829 37832380 10.1016/j.jns.2023.120829

[16] Harpsoe MC, Basit S, Andersson M, Nielsen NM, Frisch M, Wohlfahrt J, Nohr EA, Linneberg A, Jess T (2014) Body mass index and risk of autoimmune diseases: a study within the Danish National Birth Cohort. Int J Epidemiol 43:843–855 24609069 10.1093/ije/dyu045

[17] Vissing J, Atula S, Savolainen M, Mehtala J, Mehkri L, Olesen TB, Ylisaukko-Oja T, Lindberg-Schager I, Berggren F, Piehl F (2024) Epidemiology of myasthenia gravis in Denmark, Finland and Sweden: a population-based observational study. J Neurol Neurosurg Psychiatry 95:919–926 38538059 10.1136/jnnp-2023-333097 PMC11420710

[18] Park JS, Eah KY, Park JM (2022) Epidemiological profile of myasthenia gravis in South Korea using the national health insurance database. Acta Neurol Scand 145:633–640 35141872 10.1111/ane.13596

[19] Park SY, Lee JY, Lim NG, Hong YH (2016) Incidence and Prevalence of Myasthenia Gravis in Korea: A Population-Based Study Using the National Health Insurance Claims Database. J Clin Neurol 12:340–344 27165426 10.3988/jcn.2016.12.3.340 PMC4960219

[20] Feng X, Xu X, Shi Y, Liu X, Liu H, Hou H, Ji L, Li Y, Wang W, Wang Y, Li D (2019) Body Mass Index and the Risk of Rheumatoid Arthritis: An Updated Dose-Response Meta-Analysis. Biomed Res Int 2019:3579081 10.1155/2019/3579081 PMC6634074 31355257

[21] Mokry LE, Ross S, Timpson NJ, Sawcer S, Davey Smith G, Richards JB (2016) Obesity and Multiple Sclerosis: A Mendelian Randomization Study. PLoS Med 13:e1002053 27351487 10.1371/journal.pmed.1002053 PMC4924848

[22] Snekvik I, Smith CH, Nilsen TIL, Langan SM, Modalsli EH, Romundstad PR, Saunes M (2017) Obesity, Waist Circumference, Weight Change, and Risk of Incident Psoriasis: Prospective Data from the HUNT Study. J Invest Dermatol 137:2484–2490 28780086 10.1016/j.jid.2017.07.822

[23] Spatocco I, Mele G, De Rosa G, Fusco C, Ruggiero K, Pellegrini V, Carreras F, La Grotta R, Ceriello A, Procaccini C, Matarese G, Prattichizzo F, de Candia P (2026) Obesity as a Risk Factor for Autoimmune Diseases: A Systematic Review and Meta-Analysis. Obes (Silver Spring) 34:36–50 10.1002/oby.70044 PMC12724045 41186974

[24] Matarese G (2023) The link between obesity and autoimmunity. Science 379:1298–1300 36996218 10.1126/science.ade0113

[25] Dalamaga M, Chou SH, Shields K, Papageorgiou P, Polyzos SA, Mantzoros CS (2013) Leptin at the intersection of neuroendocrinology and metabolism: current evidence and therapeutic perspectives. Cell Metab 18:29–42 23770129 10.1016/j.cmet.2013.05.010

[26] Stofkova A (2009) Leptin and adiponectin: from energy and metabolic dysbalance to inflammation and autoimmunity. Endocr Regul 43:157–168 19908934

[27] Rozmilowska I, Czyzewski D, Mazur B, Adamczyk-Sowa M (2018) What is the role of adipokines in myasthenia gravis? Ther Clin Risk Manag 14:1515–1525 30214215 10.2147/TCRM.S163966 PMC6118871

[28] Miyazaki T, Hirokami Y, Matsuhashi N, Takatsuka H, Naito M (1999) Increased susceptibility of thymocytes to apoptosis in mice lacking AIM, a novel murine macrophage-derived soluble factor belonging to the scavenger receptor cysteine-rich domain superfamily. J Exp Med 189:413–422 9892623 10.1084/jem.189.2.413 PMC2192994

[29] Tobias DK, Luttmann-Gibson H, Mora S, Danik J, Bubes V, Copeland T, LeBoff MS, Cook NR, Lee IM, Buring JE, Manson JE (2023) Association of Body Weight With Response to Vitamin D Supplementation and Metabolism. JAMA Netw Open 6:e2250681 36648947 10.1001/jamanetworkopen.2022.50681 PMC9856931

[30] Yang CY, Leung PS, Adamopoulos IE, Gershwin ME (2013) The implication of vitamin D and autoimmunity: a comprehensive review. Clin Rev Allergy Immunol 45:217–226 23359064 10.1007/s12016-013-8361-3 PMC6047889

[31] Bonaccorso G (2023) Myasthenia Gravis and Vitamin D Serum Levels: A Systematic Review and Meta-analysis. CNS Neurol Disord Drug Targets 22:752–760 35796450 10.2174/1871527321666220707111344

[32] Islam MT, Moller J, Zhou X, Liang Y (2019) Life-course trajectories of body mass index and subsequent cardiovascular risk among Chinese population. PLoS ONE 14:e0223778 31600353 10.1371/journal.pone.0223778 PMC6786833

[33] Yang YC, Walsh CE, Johnson MP, Belsky DW, Reason M, Curran P, Aiello AE, Chanti-Ketterl M, Harris KM (2021) Life-course trajectories of body mass index from adolescence to old age: Racial and educational disparities. Proc Natl Acad Sci U S A 118 10.1073/pnas.2020167118 PMC8092468 33875595

[34] Baek SH, Kim JS, Jang MJ, Kim YH, Kwon O, Oh JH, Kang SY, Kang JH, Park KH, Park YS, Park KS, Shin DW, Kim BJ, Kim SM (2018) Low body mass index can be associated with the risk and poor outcomes of neuromyelitis optica with aquaporin-4 immunoglobulin G in women. J Neurol Neurosurg Psychiatry 89:1228–1230 29371414 10.1136/jnnp-2017-317202 PMC6227799

[35] Wolfe F, Michaud K (2012) Effect of body mass index on mortality and clinical status in rheumatoid arthritis. Arthritis Care Res (Hoboken) 64:1471–1479 22514152 10.1002/acr.21627

[36] Bourke CD, Berkley JA, Prendergast AJ (2016) Immune Dysfunction as a Cause and Consequence of Malnutrition. Trends Immunol 37:386–398 27237815 10.1016/j.it.2016.04.003 PMC4889773

[37] Guptill JT, Sanders DB, Evoli A (2011) Anti-MuSK antibody myasthenia gravis: clinical findings and response to treatment in two large cohorts. Muscle Nerve 44:36–40 21674519 10.1002/mus.22006

[38] Statistics Korea (KOSIS) (2026) Population dashboard of Korea. https://kosis.kr/visual/populationKorea/PopulationDashBoardMain.do. Accessed 15 Jan 2026

[39] Nelke C, Dziewas R, Minnerup J, Meuth SG, Ruck T (2019) Skeletal muscle as potential central link between sarcopenia and immune senescence. EBioMedicine 49:381–388 31662290 10.1016/j.ebiom.2019.10.034 PMC6945275

