# Supplementary material for: Association between obesity and subsequent risk of myasthenia gravis: a nationwide population-based cohort study
Source: Acta Neurol Belg. 2026 Mar 13;126(3):1095–103. doi: 10.1007/s13760-026-03022-y (PMC13415288; doi:10.1007/s13760-026-03022-y)
Supplement: Supplementary file 1 — Supplementary Material 1 [file 13760_2026_3022_MOESM1_ESM.docx]

**Supplementary Table 1. Subgroup analyses according to age and sex**

| Subgroup | | Body mass index | N | Myasthenia gravis | Duration, years | Incidence rate,  per 100,000  person-years | Hazard ratio (95% confidence interval) | |
| --- | --- | --- | --- | --- | --- | --- | --- | --- |
|  |  |  |  |  |  |  | Crude | Adjusted^*^ |
| Age^+^ | 20-64 | Underweight | 126,789 | 27 | 1,293,804.2 | 2.09 | 1.25 (0.84−1.86) | 1.41 (0.94−2.11) |
|  |  | Normal | 1,356,592 | 233 | 13,896,261.9 | 1.68 | 1 (Ref.) | 1 (Ref.) |
|  |  | Preobese | 835,446 | 159 | 8,557,458.6 | 1.86 | 1.11 (0.91−1.36) | 1.05 (0.86−1.29) |
|  |  | Obese class I | 979,405 | 202 | 10,021,282.0 | 2.02 | 1.20 (1.00−1.45) | 1.14 (0.94−1.38) |
|  |  | Obese class II | 122,648 | 39 | 1,252,515.0 | 3.11 | 1.86 (1.32−2.61) | 1.80 (1.28−2.55) |
|  | ≥65 | Underweight | 18,810 | 5 | 146,953.5 | 3.40 | 1.60 (0.63−4.08) | 1.61 (0.63−4.10) |
|  |  | Normal | 179,865 | 35 | 1,624,058.9 | 2.16 | 1 (Ref.) | 1 (Ref.) |
|  |  | Preobese | 134,484 | 37 | 1,269,729.4 | 2.91 | 1.35 (0.85−2.14) | 1.33 (0.84−2.12) |
|  |  | Obese class I | 165,986 | 53 | 1,589,970.0 | 3.33 | 1.54 (1.00−2.36) | 1.49 (0.97−2.29) |
|  |  | Obese class II | 17,189 | 9 | 164,371.4 | 5.48 | 2.53 (1.21−5.26) | 2.32 (1.11−4.85) |
|  | P for interaction | | | | | | 0.859 | 0.838 |
| Sex | Male | Underweight | 48,211 | 14 | 455,788.9 | 3.07 | 2.14 (1.23−3.75) | 2.16 (1.24−3.78) |
|  |  | Normal | 724,017 | 104 | 7,231,065.1 | 1.44 | 1 (Ref.) | 1 (Ref.) |
|  |  | Preobese | 578,289 | 92 | 5,833,314.4 | 1.58 | 1.10 (0.83−1.45) | 1.06 (0.80−1.40) |
|  |  | Obese class I | 719,654 | 129 | 7,278,053.9 | 1.77 | 1.23 (0.95−1.60) | 1.19 (0.92−1.55) |
|  |  | Obese class II | 78,013 | 21 | 789,736.7 | 2.66 | 1.85 (1.16−2.96) | 1.96 (1.22−3.14) |
|  | Female | Underweight | 97,388 | 18 | 984,968.8 | 1.83 | 0.93 (0.57−1.51) | 1.09 (0.67−1.78) |
|  |  | Normal | 812,440 | 164 | 8,289,255.7 | 1.98 | 1 (Ref.) | 1 (Ref.) |
|  |  | Preobese | 391,641 | 104 | 3,993,873.6 | 2.60 | 1.31 (1.03−1.68) | 1.14 (0.89−1.47) |
|  |  | Obese class I | 425,737 | 126 | 4,333,198.1 | 2.91 | 1.47 (1.16−1.85) | 1.21 (0.96−1.54) |
|  |  | Obese class II | 61,824 | 27 | 627,149.7 | 4.31 | 2.18 (1.45−3.27) | 1.83 (1.21−2.76) |
|  | P for interaction | | | | | | 0.084 | 0.412 |

^+^Age indicates the age at the time of the national health screening conducted in 2009.

* Adjusted for age, sex, low income, smoking, alcohol consumption, regular exercise, presence of diabetes mellitus, hypertension, dyslipidemia, and thymic disorders

**Supplementary Table 2. Crude and adjusted hazard ratios of myasthenia gravis according to waist circumference**

| Waist circumference, cm (M/F) | N | Myasthenia  gravis | Duration,  years | Incidence rate,  per 100,000  person-years | Hazard ratio (95% confidence interval) | |
| --- | --- | --- | --- | --- | --- | --- |
|  |  |  |  |  | Crude | Adjusted^*^ |
| <70 / <65 | 209,066 | 33 | 1,952,663.2 | 1.69 | 0.77 (0.56−1.06) | 0.87 (0.63−1.20) |
| 70-79 / 65-74 | 1,238,254 | 206 | 11,444,444.1 | 1.80 | 0.82 (0.71−0.96) | 0.87 (0.75−1.02) |
| 80-89 / 75-84 | 1,696,939 | 344 | 15,708,218.9 | 2.19 | 1 (Ref.) | 1 (Ref.) |
| 90-99 / 85-94 | 668,539 | 167 | 6,185,185.3 | 2.70 | 1.24 (1.05−1.46) | 1.17 (0.99−1.38) |
| ≥100 / ≥95 | 124,416 | 49 | 1,147,538.3 | 4.27 | 1.96 (1.50−2.56) | 1.82 (1.39−2.38) |
| <90 / <85 | 3,144,259 | 583 | 29,105,326.2 | 2.00 | 1 (Ref.) | 1 (Ref.) |
| ≥90 / ≥85 | 792,955 | 216 | 10,711,078.6 | 2.94 | 1.47 (1.28−1.69) | 1.33 (1.15−1.54) |

* Adjusted for age, sex, low income, smoking, alcohol consumption, regular exercise, presence of diabetes mellitus, hypertension, dyslipidemia, and thymic disorders
